# Internet addiction as a predictor of emotional eating in young adults: the incremental role of psychological variables

**DOI:** 10.3389/fpsyg.2026.1832842

**Published:** 2026-05-21

**Authors:** Hande Öngün Yılmaz, Ahmet Murat Günal, Salim Yılmaz

**Affiliations:** 1 Department of Nutrition and Dietetics, Faculty of Health Sciences, Bandırma Onyedi Eylul University, Balıkesir, Türkiye; 2 Department of Nutrition and Dietetics, Faculty of Health Sciences, Haliç University, Istanbul, Türkiye; 3 Department of Health Management, Faculty of Health Sciences, Acibadem Mehmet Ali Aydınlar University, Istanbul, Türkiye; 4 Department of Health Management, Graduate School of Health Sciences, Acibadem Mehmet Ali Aydınlar University, Istanbul, Türkiye

**Keywords:** depression, eating behavior, emotional eating, happiness, internet addiction, young adults

## Abstract

**Background:**

This study aimed to investigate factors associated with emotional eating, with a particular focus on internet addiction, happiness, and depression, while controlling for demographic and anthropometric variables in a large sample of young adults.

**Methods:**

A cross-sectional online survey was conducted among 1,808 young adults. Data were collected using the Emotional Eater Questionnaire (EEQ), Young’s Internet Addiction Test–Short Form (YIAT-SF), Happiness Scale (HS), and Beck Depression Inventory (BDI). Hierarchical multiple regression analysis was performed to examine the independent and incremental contributions of demographic variables, internet addiction, and psychological variables (happiness and depression) in explaining emotional eating.

**Results:**

Higher internet addiction and depression were associated with higher emotional eating, while happiness showed an inverse association. In hierarchical regression, demographic and anthropometric variables explained 8.2% of the variance (R^2^ = 0.082, F change = 23.094, *p* < 0.001). Adding internet addiction increased the explained variance to 27.8% (ΔR^2^ = 0.195, F change = 485.930, *p* < 0.001), and including psychological variables further improved the model (R^2^ = 0.310, ΔR^2^ = 0.033, F change = 42.795, *p* < 0.001). In the final model, internet addiction (β = 0.408, *p* < 0.001), BMI (β = 0.241, *p* < 0.001), depression (β = 0.194, *p* < 0.001), and gender (β = 0.185, *p* < 0.001) were significant predictors, whereas happiness was not statistically significant (β = 0.005, *p* = 0.822). The final model accounted for 31.0% of the variance in emotional eating [*F*(10, 1797) = 80.89, *p* < 0.001].

**Conclusion:**

Emotional eating appears to be associated not only with psychological distress but also with maladaptive behavioral patterns such as problematic internet use. Interventions targeting emotional eating should consider both psychological well-being and digital behavior patterns, particularly in young adults.

## Introduction

Emotional eating refers to the tendency to consume food in response to emotional states rather than physiological hunger cues. This behavior is commonly triggered by negative emotions such as stress, sadness, or anxiety and is often characterized by the consumption of highly palatable, energy-dense foods (Macht, 2008). Emotional eating has attracted considerable attention because of its potential contribution to unhealthy dietary patterns, weight gain, and obesity. Individuals who frequently engage in emotional eating may experience impaired appetite regulation and greater difficulty maintaining a healthy body weight, highlighting the importance of identifying the psychological and behavioral factors underlying this eating pattern (Konttinen, 2020; Zare et al., 2024).

Psychological distress has been consistently identified as a key determinant of emotional eating. Within affect-regulation frameworks, emotional eating is viewed as a coping strategy used to alleviate negative emotional experiences (Evers et al., 2018; Zaiser et al., 2025). In particular, depressive symptoms have been strongly associated with increased emotional eating tendencies across different populations (Konttinen et al., 2010; van Strien et al., 2016; Sedgi et al., 2025). Neurobiological evidence suggests that the consumption of highly palatable foods may temporarily reduce negative mood through activation of rewards-related neural pathways, reinforcing the link between emotional distress and food intake (Mills et al., 2020; Chang et al., 2022; Fuente González et al., 2022). As a result, individuals experiencing higher levels of depressive symptoms may be more vulnerable to engaging in emotional eating as a maladaptive coping mechanism (Jurek and Maruda, 2024).

In addition to psychological factors, modern lifestyle behaviors, such as digital technology use, may influence eating patterns. Problematic internet use or internet addiction has become increasingly prevalent, especially among young adults who spend substantial amounts of time engaging in online activities such as social media use, gaming, and video streaming (Moreno et al., 2022; Ruckwongpatr et al., 2022). Excessive internet use has been associated with several unhealthy lifestyle behaviors, including sedentary behavior, sleep disturbances, irregular eating patterns, and increased snacking (Alimoradi et al., 2019; Kożybska et al., 2022; Ning et al., 2024). Furthermore, problematic internet use has been linked to emotional dysregulation and psychological distress, both of which may increase vulnerability to maladaptive coping behaviors such as emotional eating (Park et al., 2022; Marchena-Giráldez et al., 2024).

Importantly, internet addiction was selected as the behavioral addiction construct in the present study because it is one of the most extensively studied and prevalent behavioral addictions among young adults, where problematic internet use has been consistently identified as a significant public health concern (Liu X. et al., 2025). Internet addiction is strongly associated with key psychological mechanisms such as impulsivity, rewards processing dysfunction, and emotion regulation difficulties, which are central processes in the development and maintenance of addictive behaviors (Brand et al., 2019).

Moreover, internet addiction shares core psychological and neurobiological mechanisms with other behavioral addictions, including gaming disorder, problematic smartphone use, and social media addiction, such as compulsive engagement, diminished self-control, and maladaptive affect regulation (Kuss and Griffiths, 2017; Brand et al., 2019). The inclusion of gaming disorder in the ICD-11 further supports the conceptualization of behavioral addictions as a spectrum of compulsive and impairing behaviors with shared underlying mechanisms (World Health Organization, 2019).

These shared mechanisms suggest that findings from internet addiction research may have partial theoretical relevance for other behavioral addictions; however, direct generalization should be made cautiously due to differences in behavioral context, reinforcement patterns, and usage motivations. Therefore, the present study conceptualizes internet addiction as a representative model of behavioral addiction processes rather than a universally generalizable construct.

Despite accumulating evidence indicating associations between problematic internet use, psychological well-being, and maladaptive eating behaviors, limited research has examined these variables simultaneously within a comprehensive multivariate framework. Although emotional eating has been linked to internet addiction and depressive symptoms in separate lines of research, few studies have evaluated their relative and incremental contributions while controlling for demographic and anthropometric factors. In particular, the extent to which internet addiction is associated with emotional eating beyond established correlates such as body mass index (BMI) and psychological distress remains unclear.

Therefore, the present study aimed to investigate the factors associated with emotional eating among young adults, with a particular focus on internet addiction and psychological variables (happiness and depression). Specifically, the objectives were to (i) describe the sociodemographic and psychological characteristics of the study sample, (ii) examine differences in internet addiction, happiness, and depression across emotional eating classifications, and (iii) assess the independent and incremental contributions of demographic variables, internet addiction, and psychological variables in explaining emotional eating using hierarchical multiple regression analysis.

It was hypothesized that higher levels of internet addiction and depression would be positively associated with emotional eating, whereas higher levels of happiness would be negatively associated with emotional eating. Furthermore, it was expected that internet addiction would account for additional variance in emotional eating beyond demographic factors, underscoring its potential relevance in understanding emotional eating patterns.

## Materials and methods

### Study design and setting

We conducted a cross-sectional, web-based survey among young adults living in Türkiye between November 18, 2025, and December 31, 2025. Recruitment used convenience and snowball sampling via social media platforms (WhatsApp, Instagram, X/Twitter, Facebook) and a Google Forms questionnaire. Given the use of convenience and snowball sampling via social media platforms accessible to the researchers, the sample may not fully represent the broader adult population in Türkiye, which should be considered when interpreting the results.

### Participants and eligibility

The population of the study consisted of adults aged 18–30 living in Türkiye. The priori power analysis (G*Power 3.1) indicated that a minimum of 1,628 participants would be required to detect a small effect size (f^2^ = 0.02) with 99% power at α = 0.05 in a multiple regression model including 10 predictors. Considering the possibility of missing data within the scope of the research, it was aimed to reach 1790 people by taking 10% more (Faul et al., 2007, 2009). The research group was determined using convenience and snowball sampling, a non-probability sampling method. Young adults who were reached through social media and group communication applications who completed the online survey by accepting to participate in the research were included in the study. In this study, among the 1920 individuals included in the study, the data of 1808 individuals were evaluated by excluding those who were missing data after data checking (*n* = 47), age < 18 years (*n* = 9) and age > 30 years (*n* = 34) diagnosed with psychiatric illness (*n* = 9), and/or following a special diet with a dietitian (*n* = 5), and/or having food allergies or intolerances (*n* = 3), and/or being pregnant or breastfeeding (*n* = 5). *Post hoc* power analysis (G*Power 3.1) indicated that the final model (R^2^ = 0.310; f^2^ = 0.449) achieved a statistical power of 1.00 with 10 predictors and a sample size of 1,808 (α = 0.05).

### Ethics

The study protocol complied with the Declaration of Helsinki and received approval from the Bandırma Onyedi Eylül University Non-Interventional Health Sciences Research Ethics Committee (Date: 17.11.2025, Application No: 2025-206). Before proceeding, participants viewed an information page explaining the study’s purpose and procedures and provided electronic informed consent. All survey responses were collected anonymously, stored on password-protected systems accessible only to the research team, and analyzed in de-identified form.

### Data collection

Prior to data collection, individuals were informed about the purpose and scope of the study, and the those included in the study were asked to approve the informed consent form at the beginning of the online survey form. The data of the study were collected online with a questionnaire form consisting of five sections. The first part of the questionnaire included the Descriptive Information Form, the second part included the Emotional Eater Questionnaire (EEQ), the third part included the Young’s Internet Addiction Test-Short Form (YIAT-SF), the fourth part included the Happiness Scale (HS), and the fifth part included the Beck Depression Inventory (BDI).

Descriptive Information Form: Age, gender, education level (literate, primary school, middle school, high school, university), marital status, employment status (employed, unemployed, student, retired, homemaker), perceived income adequacy (income less than expenses, income equal to expenses, income greater than expenses), height (cm), and weight (kg) were self-reported. Body mass index (BMI) was calculated as weight (kg) divided by height squared (m^2^).

Emotional Eater Questionnaire (EEQ): The Emotional Eater Questionnaire was developed by Garaulet et al. (2012) and adapted into Turkish by Arslantaş et al. (2019), who conducted its validity and reliability study. The scale consists of 10 items and three subdimensions: disinhibition (inability to resist eating), type of food, and guilt. Items are rated on a 4-point Likert scale (“0” = Never, “1” = Sometimes, “2” = Generally, “3” = Always). There are no reverse-scored items. Total scores range from 0 to 30, with higher scores indicating higher levels of emotional eating (Arslantaş et al., 2019). Garaulet et al. (2012) categorized scores as follows: 0–5 = non-emotional eater; 6–10 = low-level emotional eater; 11–20 = emotional eater; and 21–30 = high-level emotional eater. In the present study, Cronbach’s alpha coefficient for the EEQ was 0.912.

Young’s Internet Addiction Test-Short Form (YIAT-SF): The Young’s Internet Addiction Test was originally developed by Young (1998) and later shortened by Pawlikowski et al. (2013). The Turkish validity and reliability study of the short form was conducted by Kutlu et al. (2016). The YIAT-SF is a unidimensional scale consisting of 12 items rated on a 5-point Likert scale (1 = Never, 5 = Very often). There are no reverse-scored items. Total scores range from 12 to 60, with higher scores indicating higher levels of internet addiction (Kutlu et al., 2016). In this study, Cronbach’s alpha for the YIAT-SF was 0.948.

Happiness Scale (HS): The Happiness Scale was developed by Demirci and Ekşi (2018). It is a unidimensional instrument consisting of 6 items rated on a 5-point Likert scale. The scale contains no reverse-scored items. Total scores range from 6 to 30, with higher scores indicating higher levels of happiness (Demirci and Ekşi, 2018). In the present study, Cronbach’s alpha for the HS was 0.938.

Beck Depression Inventory (BDI): The Beck Depression Inventory was developed by Beck et al. (1961) to assess the risk of depression and the severity of depressive symptoms. The scale consists of 21 items rated on a 4-point Likert scale (0–3). Total scores range from 0 to 63. Depression severity is interpreted as follows: 0–9 minimal, 10–16 mild, 17–29 moderate, and 30–63 severe. The Turkish validity and reliability study was conducted by Hisli (1989). In this study, Cronbach’s alpha for the BDI was 0.908.

### Statistical analyses

All statistical analyses were conducted using IBM SPSS Statistics (Version 25) and R (R Core Team, 2025) with the lmtest (Zeileis and Hothorn, 2002), sandwich (Zeileis, 2004; Zeileis et al., 2020), and car (Fox and Weisberg, 2018) packages. Descriptive statistics were calculated for all study variables; continuous variables were presented as mean and standard deviation, and categorical variables as frequency and percentage. Internal consistency was evaluated using Cronbach’s alpha. Normality was assessed via skewness and kurtosis values, with values within ±3 considered acceptable (Kline, 2023). Independent samples *t*-tests were used to examine gender differences in study variables. Group differences according to emotional eating classification were examined using one-way ANOVA; where Levene’s test indicated a violation of homogeneity of variances, Games–Howell *post hoc* comparisons were used. Pearson correlation analysis was conducted to examine bivariate relationships among continuous variables. Hierarchical multiple regression analysis was performed to examine predictors of emotional eating. Variables were entered in three blocks: (1) demographic and anthropometric variables, (2) internet addiction, and (3) psychological variables. Model fit was evaluated using R^2^ and ΔR^2^ statistics. Regression assumptions were assessed as follows: multicollinearity via tolerance and VIF values, residual normality via standardized residual diagnostics, linearity via the Ramsey RESET test, and homoscedasticity via the Breusch–Pagan test. Heteroscedasticity-consistent (HC3) standard errors were computed to evaluate the robustness of OLS estimates. Statistical significance was set at *p* < 0.05.

## Results

The mean age of participants was 22.20 years (SD = 2.73), and 61.7% were female. The majority were university graduates (87.2%), single (93.6%), and students (64.1%). The mean BMI was 22.44 kg/m^2^ (SD = 3.59). Descriptive characteristics of the participants are presented in Table 1.

**TABLE 1:** Descriptive characteristics of participants (*N* = 1808).

Characteristic	Value
Age (years), mean ± SD	22.20 ± 2.73
Gender, n (%)	
Male	693 (38.3)
Female	1115 (61.7)
Education, n (%)	
Literate	8 (0.4)
Primary school	2 (0.1)
Middle school	23 (1.3)
High school	199 (11.0)
University	1526 (87.2)
Marital status, n (%)	
Single	1693 (93.6)
Married	115 (6.4)
Employment status, n (%)	
Employed	431 (23.8)
Unemployed	197 (10.9)
Student	1159 (64.1)
Retired	3 (0.2)
Homemaker	18 (1.0)
Perceived income, n (%)	
Income < expenses	380 (21.0)
Income = expenses	1032 (57.1)
Income > expenses	396 (21.9)
Height (cm), mean ± SD	170.46 ± 9.15
Weight (kg), mean ± SD	65.61 ± 13.82
BMI (kg/m^2^), mean ± SD	22.44 ± 3.59

BMI, body mass index. Perceived income refers to self-reported adequacy of income relative to expenses.

Table 2 presents the mean scores of the study scales and the categorical distribution of emotional eating and depression risk. The mean EEQ score was 9.59 (SD = 6.38). According to EEQ classifications, 29.2% of participants were classified as not emotional eaters, 34.4% as low-level emotional eaters, 31.4% as emotional eaters, and 5.0% as high-level emotional eaters. The mean YIAT-SF score was 26.06 (SD = 10.59), the mean HS score was 21.72 (SD = 5.37), and the mean BDI score was 11.64 (SD = 10.29). Based on BDI categories, 49.9% of participants were in the minimum range, 22.6% in the low range, 21.1% in the moderate range, and 6.4% in the severe range for depression risk.

**TABLE 2:** Mean scores of the Emotional Eater Questionnaire, Young’s Internet Addiction Test-Short Form, Happiness Scale, Beck Depression Inventory of the research group (*N* = 1808).

Scale	*n* (%)	Mean ± SD
EEQ		9.59 ± 6.38
EEQ classification		
Not emotional eater (0–5 points)	528 (29.2)	26.06 ± 10.59
Low-level emotional eater (6–10 points)	622 (34.4)	
Emotional eater (11–20 points)	567 (31.4)	
High-level emotional eater (21–30 points)	91 (5.0)	
YIAT-SF		
HS		21.72 ± 5.37
BDI		11.64 ± 10.29
BDI categories		
Minimum risk of depression (0–9 points)	903 (49.9)	
Low risk of depression (10–16 points)	408 (22.6)	
Moderate risk of depression (17–29 points)	381 (21.1)	
Severe risk of depression (30–63 points)	116 (6.4)	

EEQ, Emotional Eater Questionnaire; YIAT-SF, Young’s Internet Addiction Test-Short Form; HS, Happiness Scale; BDI, Beck Depression Inventory.

Prior to the main analyses, distributional properties of continuous variables were examined. Skewness and kurtosis values were as follows: YIAT-SF (skewness = 0.743, kurtosis = 0.087), HS (skewness = −0.456, kurtosis = 0.009), BDI (skewness = 1.193, kurtosis = 1.863), EEQ (skewness = 0.715, kurtosis = 0.393), BMI (skewness = 1.042, kurtosis = 2.097), and age (skewness = 1.090, kurtosis = 0.799). All values fell within the ±3 criterion, and parametric tests were deemed appropriate given the large sample size (*N* = 1,808). The reliability coefficients were 0.912 for the EEQ, 0.948 for the YIAT-SF, 0.938 for the HS, and 0.908 for the BDI, indicating excellent internal consistency for all instruments.

Table 3 presents the mean scores differed by gender. Females had significantly higher emotional eating (EEQ: 10.06 ± 6.55 vs. 8.83 ± 6.04; *t* = −3.99, *p* < 0.001) and depression scores (BDI: 12.28 ± 10.16 vs. 10.61 ± 10.43; *t* = −3.37, *p* = 0.001) compared to males. In contrast, males had significantly higher internet addiction scores (YIAT-SF: 27.08 ± 11.02 vs. 25.43 ± 10.27; *t* = 3.23, *p* = 0.001). No significant gender difference was observed in happiness scores (HS: 21.79 ± 5.25 vs. 21.60 ± 5.57; *t* = −0.72, *p* = 0.471).

**TABLE 3:** Mean scores of emotional eating, internet addiction, happiness, and depression by gender (*N* = 1808).

Scale total score	Female (*n* = 1115)	Male (*n* = 693)	t	df	*p*
EEQ	10.06 ± 6.55	8.83 ± 6.04	−3.99	1806	<0.001***
YIAT-SF	25.43 ± 10.27	27.08 ± 11.02	3.23	1806	0.001**
HS	21.79 ± 5.25	21.60 ± 5.57	−0.72	1806	0.471
BDI	12.28 ± 10.16	10.61 ± 10.43	−3.37	1806	0.001**

EEQ, Emotional Eater Questionnaire; YIAT-SF, Young’s Internet Addiction Test-Short Form; HS, Happiness Scale; BDI, Beck Depression Inventory. Values are presented as Mean ± SD. Independent samples *t*-tests.

*p < 0.05, **p < 0.01, ***p < 0.001.

Table 4 presents the mean scores of internet addiction, happiness, and depression according to emotional eating classification. Group comparisons were conducted using one-way ANOVA. YIAT-SF scores increased progressively across emotional eating groups, from 20.62 ± 8.93 in not emotional eaters to 37.09 ± 13.99 in high-level emotional eaters (*F* = 131.803, *p* < 0.001). The assumption of homogeneity of variances was tested using Levene’s test, which indicated violations for internet addiction (YIAT-SF), *F*(3, 1804) = 26.63, *p* < 0.001, happiness (HS), *F*(3, 1804) = 7.59, *p* < 0.001, and depression (BDI), *F*(3, 1804) = 19.40, *p* < 0.001. Therefore, Games–Howell *post hoc* comparisons were conducted.

**TABLE 4:** Mean scores of the internet addiction, happiness, and depression according to emotional eating classification (*N* = 1808).

Scale total score	EEQ classification						
	Not emotional eater^a^	Low-level emotional eater^b^	Emotional eater^c^	High-level emotional eater^d^	F	*p*	*Post hoc*
YIAT-SF	20.62 ± 8.93	25.35 ± 8.81	30.14 ± 10.16	37.09 ± 13.99	131.803	<0.001	a < b, a < c, a < d, b < c, b < d, c < d
HS	22.62 ± 5.99	21.81 ± 5.03	20.67 ± 4.82	22.43 ± 6.04	12.997	<0.001	a > c, b > c, d > c
BDI	8.59 ± 9.05	10.55 ± 8.86	13.97 ± 10.22	22.31 ± 15.48	66.571	<0.001	a < b, a < c, a < d, b < c, b < d, c < d

EEQ, Emotional Eater Questionnaire; YIAT-SF, Young’s Internet Addiction Test-Short Form; HS, Happiness Scale; BDI, Beck Depression Inventory. Values are presented as Mean ± SD.

*^a^*Not emotional eater: EEQ score 0–5 (*n* = 528);

*^b^*Low-level emotional eater: EEQ score 6–10 (*n* = 622);

*^c^*Emotional eater: EEQ score 11–20 (*n* = 567);

*^d^*High-level Emotional eater: EEQ score 21–30 (*n* = 91). One-way ANOVA with Games–Howell *post-hoc* test was used for group comparisons because Levene’s test indicated a violation of homogeneity of variances (*p* < 0.001). The *Post hoc* column shows significant pairwise comparisons (*p* < 0.05).

*Post hoc* analyses revealed significant differences between all groups for YIAT-SF scores (a < b, a < c, a < d, b < c, b < d, c < d). HS scores differed significantly according to emotional eating classification (*F* = 12.997, *p* < 0.001), with emotional eaters showing the lowest happiness scores (20.67 ± 4.82). Games–Howell comparisons indicated that emotional eaters scored significantly lower than not emotional eaters, low-level emotional eaters, and high-level emotional eaters (a > c, b > c, d > c). BDI scores increased with emotional eating, ranging from 8.59 ± 9.05 in not emotional eaters to 22.31 ± 15.48 in high-level emotional eaters (*F* = 66.571, *p* < 0.001), and Games–Howell *post-hoc* tests demonstrated significant differences between all groups (a < b, a < c, a < d, b < c, b < d, c < d).

Table 5 presents the Pearson correlation coefficients among emotional eating, internet addiction, happiness, depression, body mass index, and age. Emotional eating was positively correlated with internet addiction (*r* = 0.447, *p* < 0.001), depression (*r* = 0.304, *p* < 0.001), and BMI (*r* = 0.217, *p* < 0.001). No significant association was observed between emotional eating and age (*r* = −0.014, *p* = 0.560). Emotional eating was negatively correlated with happiness (*r* = −0.090, *p* < 0.001).

**TABLE 5:** Pearson correlation matrix for emotional eating, internet addiction, happiness, depression, body mass index, and age (*N* = 1808).

Variable	1	2	3	4	5	6
1. EEQ	–	–	–	–	–	–
2. YIAT-SF	0.447***					
3. HS	−0.090***	−0.057*				
4. BDI	0.304***	0.230***	−0.374***			
5. BMI	0.217***	0.054*	−0.017	0.045		
6. Age	−0.014	−0.093***	0.047*	−0.112***	0.200***	

EEQ, Emotional Eater Questionnaire; YIAT-SF, Young’s Internet Addiction Test-Short Form; HS, Happiness Scale; BDI, Beck Depression Inventory; BMI, body mass index. Pearson correlation analysis was performed to examine the relationships among continuous variables. *p < 0.05, **p < 0.01, ***p < 0.001 (two-tailed).

Internet addiction showed a positive correlation with depression (*r* = 0.230, *p* < 0.001) and BMI (*r* = 0.054, *p* < 0.05), and a negative correlation with happiness (*r* = −0.057, *p* < 0.05) and age (*r* = −0.093, *p* < 0.001). Happiness was negatively associated with depression (*r* = −0.374, *p* < 0.001) and positively associated with age (*r* = 0.047, *p* < 0.05). Depression was negatively correlated with age (*r* = −0.112, *p* < 0.001). BMI was positively correlated with age (*r* = 0.200, *p* < 0.001).

Table 6 presents the hierarchical multiple regression analysis examining predictors of emotional eating (EEQ total score).

**TABLE 6:** Hierarchical multiple regression analysis predicting emotional eating (EEQ total score) (*N* = 1808).

Variable	B	SE	β	t	*p*	95% CI	Tolerance	VIF	R^2^	Adj. R^2^	ΔR^2^	F change
(Intercept)	0.304	2.053	−0.040	0.148	0.882	[−3.723, 4.330]	0.578	1.731	0.082	0.079	0.082***	23.094***
Block 1: Demographic	−0.093	0.070		−1.339	0.181	[−0.230, 0.043]						
Age									0.278	0.274	0.195***	485.930***
Gender (female)	2.287	0.316	0.174	7.240	<0.001***	[1.668, 2.907]	0.881	1.136				
Education (university)	−0.299	0.446	−0.016	−0.672	0.502	[−1.174, 0.575]	0.935	1.070				
Marital status (single)	−0.322	0.670	−0.012	−0.481	0.631	[−1.636, 0.992]	0.777	1.287				
Employment (employed)	−0.431	0.407	−0.029	−1.058	0.290	[−1.229, 0.368]	0.690	1.450				
Income (middle/high)	−0.737	0.356	−0.047	−2.071	0.039*	[−1.436, −0.039]	0.987	1.014				
BMI (kg/m^2^)	0.499	0.043	0.281	11.519	<0.001***	[0.414, 0.583]	0.859	1.164				
(Intercept)	−7.893	1.860		−4.244	<0.001***	[−11.541, −4.246]						
Block 2: + Internet Addiction												
Age	−0.039	0.062	−0.016	−0.625	0.532	[−0.160, 0.083]	0.577	1.733	0.310	0.307	0.033***	42.795***
Gender (female)	2.721	0.281	0.207	9.682	<0.001***	[2.170, 3.272]	0.876	1.141				
Education (university)	0.030	0.396	0.002	0.076	0.940	[−0.746, 0.806]	0.934	1.071				
Marital status (single)	−0.459	0.595	−0.018	−0.773	0.440	[−1.626, 0.707]	0.777	1.287				
Employment (employed)	0.144	0.362	0.010	0.399	0.690	[−0.566, 0.855]	0.686	1.457				
Income (middle/high)	−0.358	0.317	−0.023	−1.131	0.258	[−0.979, 0.263]	0.984	1.017				
BMI (kg/m^2^)	0.458	0.038	0.258	11.907	<0.001***	[0.383, 0.533]	0.857	1.166				
YIAT-SF	0.270	0.012	0.448	22.044	<0.001***	[0.246, 0.294]	0.973	1.028				
(Intercept)	−8.208	1.876		−4.375	<0.001***	[−11.887, −4.529]						
Block 3: + Psychological Variables												
Age	−0.040	0.060	−0.017	−0.669	0.504	[−0.159, 0.078]	0.577	1.734				
Gender (female)	2.435	0.277	0.185	8.793	<0.001***	[1.892, 2.978]	0.863	1.159				
Education (university)	0.370	0.390	0.019	0.947	0.344	[−0.396, 1.135]	0.918	1.089				
Marital status (single)	−0.861	0.584	−0.033	−1.475	0.140	[−2.006, 0.284]	0.770	1.298				
Employment (employed)	0.427	0.356	0.029	1.202	0.229	[−0.270, 1.125]	0.681	1.468				
Income (middle/high)	0.032	0.313	0.002	0.101	0.920	[−0.582, 0.645]	0.962	1.039				
BMI (kg/m^2^)	0.429	0.038	0.241	11.362	<0.001***	[0.355, 0.503]	0.851	1.175				
YIAT-SF	0.246	0.012	0.408	20.035	<0.001***	[0.222, 0.270]	0.927	1.079				
HS	0.006	0.025	0.005	0.224	0.822	[−0.044, 0.055]	0.843	1.186				
BDI	0.120	0.014	0.194	8.706	<0.001***	[0.093, 0.147]	0.775	1.290				

Hierarchical multiple regression analysis was conducted to examine factors associated with emotional eating (EEQ total score). Variables were entered in three sequential blocks: (1) demographic and anthropometric variables (age, gender, education, marital status, employment status, income level, BMI), (2) internet addiction (YIAT-SF), and (3) psychological variables (happiness and depression). Categorical variables were dummy coded prior to analysis. Reference categories = Male; non-university; married; non-employed; low income. Multicollinearity assumptions were satisfied, with tolerance values ranging from 0.577 to 0.987 and VIF values ranging from 1.014 to 1.734. Model fit was evaluated using R^2^ and ΔR^2^ values. Statistical significance was set at *p* < 0.05. Independence of observations was assumed given the cross-sectional study design. Final model: R^2^ = 0.310, Adjusted R^2^ = 0.307, *F*_(10,1797)_ = 80.89, *p* < 0.001. CI, confidence interval; BMI, body mass index; EEQ, Emotional Eater Questionnaire; YIAT-SF, Young’s Internet Addiction Test-Short Form; HS, Happiness Scale; BDI, Beck Depression Inventory. *p < 0.05, **p < 0.01, ***p < 0.001.

In Block 1, demographic and anthropometric variables (age, gender, education, marital status, employment status, income level, and BMI) were entered into the model. The model was statistically significant, *F*_(7, 1800)_ = 23.09, *p* < 0.001, explaining 8.2% of the variance in emotional eating (R^2^ = 0.082, Adjusted R^2^ = 0.079). Female gender (*B* = 2.287, β = 0.174, *p* < 0.001), lower income (middle/high coded as 1; *B* = −0.737, β = −0.047, *p* = 0.039), and higher BMI (*B* = 0.499, β = 0.281, *p* < 0.001) were significantly associated with higher emotional eating scores. Age, education, marital status, and employment status were not significant predictors.

In Block 2, internet addiction (YIAT-SF) was added to the model. This addition significantly improved model fit [ΔR^2^ = 0.195, ΔF_(1, 1799)_ = 485.93, *p* < 0.001], increasing the explained variance to 27.8% (R^2^ = 0.278, Adjusted R^2^ = 0.274), *F*_(8, 1799)_ = 86.39, *p* < 0.001. Internet addiction emerged as a strong positive predictor of emotional eating (*B* = 0.270, β = 0.448, *p* < 0.001). Female gender and BMI remained significant, whereas income was no longer significant.

In Block 3, psychological variables (happiness and depression) were included. This block produced a further significant increase in explained variance [ΔR^2^ = 0.033, ΔF_(2, 1797)_ = 42.80, *p* < 0.001]. The final model accounted for 31.0% of the variance in emotional eating (R^2^ = 0.310, Adjusted R^2^ = 0.307), *F*_(10, 1797)_ = 80.89, *p* < 0.001. In the final model, female gender (*B* = 2.435, β = 0.185, *p* < 0.001), BMI (*B* = 0.429, β = 0.241, *p* < 0.001), internet addiction (*B* = 0.246, β = 0.408, *p* < 0.001), and depression (*B* = 0.120, β = 0.194, *p* < 0.001) remained significant predictors, whereas happiness and other demographic variables were not significant. Multicollinearity diagnostics indicated no serious concerns (Tolerance = 0.577–0.987; VIF = 1.014–1.734). Standardized residuals had a mean of 0.00 (SD = 0.997) and ranged between −3.716 and 4.248, indicating an approximately normal distribution of residuals. The Ramsey RESET test indicated no evidence of model misspecification [*F*_(2, 1795)_ = 2.208, *p* = 0.110], supporting the appropriateness of the linear functional form. The Breusch–Pagan test indicated the presence of heteroscedasticity (BP = 65.354, df = 10, *p* < 0.001). However, when the model was re-estimated using robust (HC3) standard errors, the results remained unchanged, and all predictors retained their statistical significance. This suggests that the results are robust despite this violation.

The final model explained 31.0% of the variance in emotional eating [*F*_(10, 1797)_ = 80.89, *p* < 0.001]. Internet addiction emerged as the strongest predictor (β = 0.408, *p* < 0.001), followed by BMI (β = 0.241, *p* < 0.001) and depressive symptoms (β = 0.194, *p* < 0.001), while happiness was not significant. These findings underscore the prominent role of problematic internet use and depressive symptoms in emotional eating beyond demographic factors.

## Discussion

The present study investigated the associations between emotional eating, internet addiction, happiness, and depressive symptoms among young adults in Türkiye using a hierarchical regression framework. The findings demonstrate that emotional eating is associated with a combination of behavioral, psychological, and demographic factors. Among all variables examined, internet addiction emerged as the strongest predictor of emotional eating, even after controlling for demographic characteristics and body mass index. Depressive symptoms and BMI were also significant predictors, while happiness did not independently predict emotional eating in the final model. These findings suggest that emotional eating in young adults may be influenced not only by psychological distress but also by maladaptive digital behavior patterns.

One of the most notable findings of this study is the strong positive association between internet addiction and emotional eating. In the hierarchical regression model, the inclusion of internet addiction substantially increased the explained variance in emotional eating, indicating that problematic internet use may represent an important behavioral correlate beyond traditional demographic and anthropometric factors. This finding is consistent with a growing body of research demonstrating that excessive internet use is associated with a variety of unhealthy lifestyle behaviors, including irregular eating patterns, increased snacking, and a higher intake of energy-dense foods (Gümüş et al., 2023; Liu X. et al., 2025). For example, previous research among university students has shown that higher levels of internet addiction are linked to poorer eating behaviors and reduced adaptive eating patterns such as intuitive eating (Liu K. et al., 2025). Similarly, studies focusing on social media addiction have reported significant associations between excessive social media use and maladaptive eating behaviors, including emotional and external eating (Göbel et al., 2023; Gümüş et al., 2023). These findings support the notion that digital lifestyle patterns may increasingly influence eating behaviors among young adults.

It is important to note that the Turkish version of the Young Internet Addiction Test–Short Form does not provide a validated clinical cut-off score (Kutlu et al., 2016); therefore, the present findings should not be interpreted in terms of diagnostic classification, and it is not possible to determine the proportion of individuals exceeding a pathological threshold. Instead, internet addiction was conceptualized and analyzed as a continuous construct reflecting a severity spectrum ranging from low to high levels of problematic use (Andreassen, 2015). From this perspective, the observed positive association between emotional eating and internet addiction indicates a graded increase in maladaptive internet use behaviors rather than the presence of clinically defined addiction cases. This approach is consistent with the widely accepted view in the literature that problematic internet use is better conceptualized as a dimensional construct rather than a categorical disorder, involving varying degrees of compulsivity, impaired control, and functional impairment (Kuss and Griffiths, 2017; Brand et al., 2019). Accordingly, problematic internet use shares core psychological and neurobehavioral mechanisms with other behavioral addictions, including rewards sensitivity and deficits in emotion regulation, supporting its conceptualization along a continuum of severity rather than a dichotomous diagnosis (Fineberg et al., 2018; Brand et al., 2019). Therefore, the results should be interpreted as reflecting increasing risk levels of problematic internet use rather than definitive clinical diagnoses. Future research using instruments with established clinical thresholds may provide more precise estimates regarding the prevalence of clinically significant levels of problematic internet use.

Young adults who spend extended periods online may also be more likely to engage in distracted or mindless eating. Screen-based activities such as social media use, gaming, or video streaming often occur simultaneously with food consumption, which may reduce awareness of internal hunger and satiety cues and promote overeating (Göbel et al., 2023). Distracted eating has been widely recognized as a factor contributing to impaired dietary self-regulation, as individuals who eat while engaged in other activities tend to consume larger amounts of food and experience reduced memory of their food intake (Robinson et al., 2013). Over time, such patterns may reinforce emotional or impulsive eating behaviors, particularly in environments characterized by constant digital engagement.

Several mechanisms may explain the observed relationship between internet addiction and emotional eating. First, problematic internet use may function as a maladaptive coping strategy for managing negative emotions or psychological stress. Behavioral addiction models suggest that individuals may rely on digital activities to escape from distress or regulate emotional states, which may coexist with other maladaptive coping strategies such as emotional eating (Kuss and Griffiths, 2017). In this context, both excessive internet use and emotional eating may serve similar regulatory functions by providing short-term emotional relief. Second, prolonged screen exposure often promotes sedentary behavior and increases opportunities for frequent snacking, particularly during activities such as watching videos, gaming, or browsing social media. Evidence suggests that screen-based or distracted eating is associated with increased food intake, particularly of energy-dense foods, partly due to the context in which eating occurs (Robinson et al., 2013). Such contexts may encourage automatic or impulsive eating behaviors that are less responsive to physiological hunger signals. Third, excessive internet use is frequently associated with disrupted sleep patterns, which may further influence appetite regulation. Evidence suggests that problematic internet use is linked to insomnia and poor sleep quality among young adults (Yao et al., 2024). Sleep disruption may alter appetite-regulating hormones such as ghrelin and leptin, potentially increasing hunger and preference for high-calorie foods. In addition, sleep deprivation has been associated with increased emotional reactivity and reduced impulse control, both of which may increase vulnerability to emotional eating (St-Onge et al., 2016). Taken together, these behavioral, psychological, and physiological mechanisms provide plausible explanations for the observed relationship between internet addiction and emotional eating.

In addition to internet addiction, depressive symptoms were significantly associated with emotional eating. This finding is consistent with literature indicating that negative affect and depressive mood are important psychological drivers of emotional eating. Previous studies have consistently shown that individuals experiencing depressive symptoms are more likely to eat in response to emotional distress rather than physiological hunger cues (Konttinen, 2020; Jurek and Maruda, 2024). Emotional eating has therefore been widely conceptualized as a maladaptive coping strategy in which food consumption serves as a temporary means of regulating negative emotional states.

Depressive symptoms may intensify this pattern by increasing vulnerability to rewards-seeking behaviors, including the consumption of highly palatable foods rich in sugar and fat. Experimental and observational research suggests that individuals with higher levels of depressive symptoms tend to prefer energy-dense comfort foods, which may provide short-term emotional relief (Fuente González et al., 2022; Sedgi et al., 2025). These foods are thought to activate rewards-related neural pathways involving dopaminergic signaling, temporarily improving mood and reducing psychological discomfort (Ljubičić et al., 2023). However, repeated reliance on food for emotional regulation may reinforce the behavioral association between negative emotions and food intake over time (Macht, 2008). Neurobiological explanations further support this mechanism. Palatable foods can stimulate rewards systems in the brain, particularly within the mesolimbic dopamine pathway, which plays a central role in motivation and reinforcement (Mills et al., 2020). Activation of these pathways may temporarily alleviate negative mood states, thereby strengthening the learned association between emotional distress and eating behavior. In addition, depressive symptoms are often accompanied by alterations in stress-related neuroendocrine processes, including dysregulation of the hypothalamic–pituitary–adrenal (HPA) axis. Chronic activation of this stress system has been linked to increased appetite and a greater preference for highly palatable foods, further contributing to emotional eating behaviors (Chang et al., 2022; Günal, 2023).

Taken together, these psychological and neurobiological mechanisms suggest that individuals experiencing higher levels of depressive symptoms may be particularly vulnerable to emotional eating as a form of mood regulation. This pattern may create a cyclical relationship in which emotional distress promotes maladaptive eating behaviors, which in turn may contribute to further psychological discomfort and negative affect.

Body mass index (BMI) also emerged as a significant predictor of emotional eating in the present study. Higher BMI values were associated with higher emotional eating scores, supporting previous findings that individuals with greater body weight are more likely to report emotional eating behaviors. Research indicates that emotional eating is positively associated with overweight and obesity, suggesting that individuals with higher BMI may be more susceptible to emotion-driven food consumption patterns (Konttinen et al., 2010; Öngün Yılmaz et al., 2024). For instance, large-scale observational studies have demonstrated that emotional eating tendencies are positively correlated with BMI across both men and women, although the strength of this relationship may vary depending on individual psychological and behavioral characteristics (Konttinen et al., 2010).

The relationship between BMI and emotional eating is likely to be bidirectional and multifactorial. On one hand, repeated episodes of emotional eating may lead to excessive caloric intake, particularly through the consumption of energy-dense and highly palatable foods, thereby contributing to gradual weight gain over time. Emotional eating has frequently been identified as a behavioral pathway linking psychological distress to obesity-related outcomes (Wang et al., 2023). On the other hand, individuals with higher body weight may experience greater levels of body dissatisfaction, internalized weight stigma, and social pressure related to dieting or appearance. These psychosocial stressors can increase emotional distress and may trigger maladaptive coping behaviors, including emotional eating (Öngün Yılmaz and Köse, 2020). In this context, emotional eating may function both as a contributing factor to weight gain and as a response to the psychological challenges associated with higher body weight. Such reciprocal interactions highlight the complex interplay between psychological factors, eating behavior, and body weight regulation.

Gender differences were also observed in the present study, with female participants reporting higher levels of emotional eating compared with males. This finding is consistent with literature indicating that emotional eating is generally more prevalent among women. Previous research has repeatedly shown that women tend to report higher scores on emotional eating scales and are more likely to engage in eating in response to negative emotional states (Zare et al., 2024; Zaiser et al., 2025). One possible explanation involves sociocultural influences. Women are often exposed to stronger societal pressures related to body image, thinness ideals, and dieting behaviors. These pressures may contribute to heightened body dissatisfaction and increased psychological vulnerability related to eating behaviors, which in turn may promote emotional eating patterns (van Strien et al., 2020; Günal et al., 2025).

The observed gender differences in emotional eating, internet addiction, and depression are consistent with existing literature. Higher emotional eating and depression scores among females may be explained by greater susceptibility to affect-driven eating behaviors and internalizing symptoms, which have been widely documented in prior research (Eichen et al., 2013; Salk et al., 2017). In contrast, the higher internet addiction scores observed in males align with studies suggesting that males are more prone to problematic internet use, particularly due to greater engagement in online gaming and risk-taking digital behaviors (Kuss et al., 2014; Su et al., 2019). The lack of a significant gender difference in happiness scores is also consistent with previous findings indicating that subjective well-being tends to be relatively stable across genders despite differences in specific psychological risk factors (Diener et al., 2018). Overall, these findings highlight the importance of considering gender-specific behavioral and psychological patterns when examining emotional eating and related mental health outcomes.

The findings regarding BMI and gender reinforce the importance of considering both biological and psychosocial determinants when examining emotional eating behaviors. These results suggest that interventions aimed at reducing emotional eating should incorporate weight-related psychological factors as well as gender-sensitive approaches that address emotional regulation, body image concerns, and coping strategies.

In contrast to depressive symptoms, happiness did not remain a significant predictor of emotional eating in the final regression model after controlling for other variables. Although happiness demonstrated a small negative correlation with emotional eating in the bivariate analysis, its independent association disappeared when depressive symptoms and internet addiction were included in the model. This finding suggests that the influence of positive psychological constructs may become less prominent when more proximal psychological risk factors are considered simultaneously. Previous studies examining the psychological determinants of emotional eating have similarly reported that negative emotional states tend to exert stronger and more consistent effects on maladaptive eating behaviors compared with positive affective states (van Strien et al., 2016). One possible explanation for this pattern is that negative affect plays a more central role in the motivational processes underlying emotional eating. Emotional eating is commonly conceptualized within affect-regulation frameworks, which propose that individuals consume food as a means of alleviating negative emotions such as sadness, stress, anxiety, or frustration. In this context, the presence of negative affect may act as a more immediate trigger for eating behavior than the absence of positive affect (Macht, 2008). Empirical evidence supports this view, indicating that emotional eating episodes are more frequently associated with increases in negative mood states rather than decreases in positive mood states (Evers et al., 2018). Therefore, while higher levels of happiness may be generally associated with better psychological well-being, they may not independently protect against emotional eating once depressive symptoms and other psychological stressors are considered.

Another possible explanation relates to the conceptual and statistical overlap between happiness and depressive symptoms. Positive and negative affective constructs are often moderately correlated, and measures of happiness frequently capture aspects of overall psychological well-being that are inversely related to depressive symptomatology. When both constructs are included in regression models, depressive symptoms may account for a larger proportion of variance related to emotional distress, thereby reducing the apparent independent contribution of happiness. From a methodological perspective, this phenomenon may reflect shared variance between constructs representing opposite ends of the emotional well-being spectrum (Chen et al., 2013; Cao et al., 2025).

Furthermore, previous research suggests that emotional eating is more strongly associated with psychological vulnerability factors such as depression, stress, and anxiety than with indicators of positive psychological functioning. A study has demonstrated that depressive symptoms and perceived stress consistently predict emotional eating behaviors, whereas measures of life satisfaction or happiness show weaker or inconsistent associations (Konttinen et al., 2010). This pattern highlights the possibility that maladaptive coping behaviors are primarily driven by distress-related processes rather than by the absence of positive emotional states. Taken together, the findings of the present study suggest that depressive symptoms represent a more salient psychological predictor of emotional eating than happiness when both constructs are considered simultaneously. This result underscores the importance of addressing negative emotional states in interventions aimed at reducing emotional eating behaviors. Psychological strategies focusing on depression management, emotional regulation, and stress coping may therefore be particularly relevant for preventing maladaptive eating patterns in young adults.

### Strengths and limitations

This study has several notable strengths. First, it examined multiple psychological and behavioral determinants of emotional eating including internet addiction, depressive symptoms, and happiness within a single analytical framework. Second, the use of hierarchical regression analysis allowed for the identification of the additional explanatory contribution of internet addiction beyond demographic variables. Finally, the study contributes to the emerging literature on the relationship between digital behaviors and eating patterns among young adults.

Several limitations should be considered when interpreting the findings. The cross-sectional design prevents causal or temporal conclusions regarding the relationships between internet addiction, depressive symptoms, and emotional eating. In addition, all variables were assessed using self-report measures, which may introduce recall or social desirability bias. The study sample consisted primarily of young adults, which may limit the generalizability of the findings to other age groups or populations.

### Public health and clinical implications

The findings highlight the importance of considering both psychological well-being and digital lifestyle behaviors when addressing emotional eating. Screening for depressive symptoms and problematic internet use may be beneficial in nutritional and behavioral interventions targeting maladaptive eating patterns. Integrating strategies that promote emotional regulation, healthy coping mechanisms, and balanced digital habits may help reduce the risk of emotional eating among young adults.

### Future research

Future studies should employ longitudinal designs to clarify the causal relationships between internet addiction, psychological distress, and emotional eating. Investigating potential mediating factors such as stress, sleep quality, and emotional regulation may also provide a deeper understanding of the mechanisms underlying these associations. Additionally, research involving more diverse populations and cultural contexts would help determine the generalizability of these findings.

## Conclusion

In conclusion, the present study demonstrates that internet addiction and depressive symptoms are significant predictors of emotional eating among young adults, while BMI and gender also contribute to this relationship. The findings suggest that psychological distress and digital behaviors may play an important role in shaping maladaptive eating patterns. Addressing emotional well-being alongside digital lifestyle factors may therefore be essential for developing effective strategies to prevent emotional eating.

## Data Availability

The raw data supporting the conclusions of this article will be made available by the authors, without undue reservation.
